# Supplementary material for: Activation of mitochondrial unfolded protein response protects against multiple exogenous stressors
Source: Life Sci Alliance. 2021 Sep 28;4(12):e202101182. doi: 10.26508/lsa.202101182 (PMC8500221; doi:10.26508/lsa.202101182)
Supplement: Supplementary file 5 [file LSA-2021-01182_TableS5.docx]

**Table S5. Effect of modulating ATFS-1 levels and activation on stress resistance and lifespan.**

|  | ***atfs-1(et15)***  **Gain-of-function** | ***atfs-1(et17)***  **Gain-of-function** | ***atfs-1(gk3094)***  **Loss-of-function** |
| --- | --- | --- | --- |
| Acute oxidative stress resistance | **↑** | **↑** | **↓** |
| Chronic oxidative stress resistance | **↓** | **↑** | **↓** |
| Heat stress resistance | **↓** | **=** | **↓** |
| ER stress resistance | **↑** | **↑** | **=** |
| Osmotic stress resistance | **↑** | **↑** | **↓** |
| Anoxia resistance | **↑** | **↑** | **↓** |
| Bacterial pathogens resistance (fast kill) | **↑** | **↑** | **↑** |
| Bacterial pathogens resistance (slow kill) | **==** | **↓↑** | **↑↑** |
| Lifespan | **↓** | **↓** | **=** |
